# Computerized manometry use to evaluate spasm in pharyngoesophageal segment in patients with poor tracheoesophageal speech before and after treatment with botulinum toxin

**DOI:** 10.1016/S1808-8694(15)30776-X

**Published:** 2015-10-19

**Authors:** Carlos T. Chone, Vinícius Oliveira Seixas, Nelson A. Andreollo, Elizabeth Quagliato, Irene H.K. Barcelos, Ana L. Spina, Agrício N. Crespo

**Affiliations:** 1 PhD; Professor; Otorhinolaryngologist Coordinator of the Head and Neck Department – Otorhinolaryngology Program–Unicamp; 2 Resident physician, Head and Neck–Otorhinolaryngology Program–Unicamp; 3 PhD; Professor; Gastric Surgeon – Head of the Surgery Department–Unicamp; 4 PhD; Professor; Neurologist, Department of Neurology, Unicamp; 5 PhD; Professor; Radiologist; Head of the Radiology Department–Unicamp; 6 Speech and Hearing Therapist; Graduate Student – Otorhinolaryngology and Head and Neck Program–Unicamp; 7 PhD; Professor; Otorhinolaryngologist – Head of the Otorhinolaryngology and Head and Neck Program–Unicamp

**Keywords:** toxin, laryngectomy, manometry, neck, speech

## Abstract

Tracheoesophageal voice (TEV) with voice prosthesis (VP) is an efficient and reproducible method used in vocal rehabilitation after total laryngectomy (TL), prevented by spasms in the pharyngoesophageal segment (PES). Computerized Manometry (CM) is a new, direct and objective method used to assess the PES.

**Aim:**

to carry out an objective analysis of the PES, with CM, before and after the injection of botulinum toxin (BT).

**Study design:**

clinical-prospective.

**Materials and Methods:**

analysis of eight patients consecutively submitted to TL with TEV and VP, without vocal emission, with PES spasms seen through videofluoroscopy, considered the gold standard for spasm detection. All had their spasms treated with the injection of 100 units of BT in the PES. The assessment was based on PES videofluoroscopy and CM, before and after BT injection.

**Results:**

There was a PES pressure reduction according to the CM after BT injection in all patients. The average pressure in the PES seen through the CM in eight patients before BT injection was 25.36 mmHg, and afterwards it dropped to 14.31 mmHg (p=0.004). There was vocal emission without stress and PES spasm improvement seen through the videolaryngoscopy after BT injection.

**Conclusion:**

We observed a reduction in PES pressure after BT injection, seen through CM in all the patients, with spasms improvement seen through videofluoroscopy.

## INTRODUCTION

Between 9% and 79% of the patients rehabilitated after total laryngectomy (TL) with tracheoesophageal speech (TES) and speech prosthesis (SP) after primary or secondary tracheoesophageal puncture (TEP) present stress-related speech difficulty associated to changes in the motility of the pharyngoesophageal segment (PES), secondary to its pharyngospasm^1^, ^2^, ^3^, ^4^, ^5^, ^6^, ^7^, ^8^, ^9^, ^10^, ^11^, ^12^, ^13^. This PES alteration can be treated in three different ways: myotomy of middle and lower pharynx constrictors, neurectomy of the pharyngeal plexus, and the recently published technique of chemically denervating the PES with botulinum toxin (BT)^6^, ^7^, ^8^^,^^10^^,^^11^^,^^14^, ^15^, ^16^, ^17^, ^18^, ^19^, ^20^, ^21^, ^22^, ^23^, ^24^. Botulinum toxin is a pre-synaptic blocker that prevents the release of acetylcholine in the neuromuscular junction. PES relaxation after BT application in the region can be seen through videofluoroscopy^3^^,^^4^^,^^7^^,^^15^^,^^25^. However, small variations cannot be quantified. There are indirect assessment methods that use PES pressure, as the modified insufflation test^4^^,^^6^^,^^15^, measurement of intratracheal pressure and speech time duration^7^^,^^18^. This study was developed to objectively quantify PES relaxation in spastic total laryngectomy patients after BT injection and relate it to improvements in speech quality. Esophageal manometry was used to measure PES median pressure before and after BT injection in the spastic area.

## MATERIALS AND METHOD

Eight consecutive patients seen in our institution between January of 2004 and October of 2006 with TES under stress and speech time of one second or less were included in this study. All had indwelling Blom-Singer (Inhealth®) speech prosthesis inserted after primary or secondary TEP. The patients were included in this study after at least six months of speech rehabilitation sessions. The speech rehabilitation sessions done with the total laryngectomy patients with TES and SP were conducted by one same experienced specialized therapist.

This study was approved by the ethics committee at our institution under permit 546/2005. Informed consent terms were collected from all patients participating in the study.

Tests conducted with the patients included assessment of mean speech time, acoustic analysis, swallowing and speech videofluoroscopy, 4-channel esophageal manometry with pneumocapillary infusion and computerized polygraph before and after injection of 100U of BT (Botox®) in the PES spastic area. Mean speech time was measured using a Tissot® stopwatch after three consecutive takes in which the patients were asked to utter the vowel /a/ in a prolonged manner after maximum air inhalation. Speech acoustic assessment was done at the speech lab using software package MDVP (Multidimensional Voice Program) by Kay Elemetrics Corporation.

**Figure 1:**
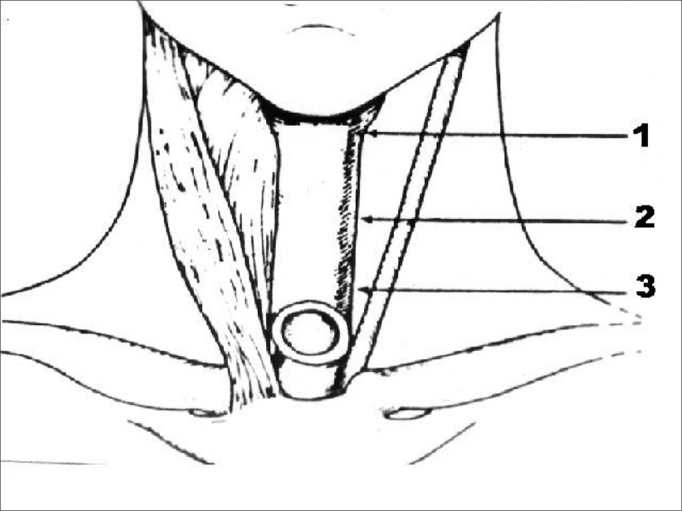
Schematic representation of areas of botulinum toxin injection on the three thirds of the pharyngoesophageal segment.

Patients were requested to utter and sustain vowel /a/. The acoustic parameter used to assess speech was presence or absence of harmonics. Speech samples were recorded with a Teac W518R recorder in chrome cassette tapes and using a Prologue microphone placed 5 centimeters from the patients' mouths. Speech samples were recorded in a soundproof booth with noise level treatment. Videofluoroscopy was considered the golden standard to diagnose PES spasm. All patients complained of dysphagia. Botulinum toxin injections were applied in each third of the PES (Fig. 1) under electromyographic control of pharyngeal constrictor muscles without local anesthesia. Pharyngeal constrictor muscle punctures were done by the author, and electromyographic tracings interpreted by one same specialist. A Compass Portabook II Nicolet electromyograph connected to a Compaq® workstation was used.

Statistical analysis done for speech time, PES pressure, presence or absence of harmonics, and PES videofluoroscopy findings before and after BT injection was done using the Binomial test. A significance level of 0.05 was considered.

## RESULTS

**Figure 2:**
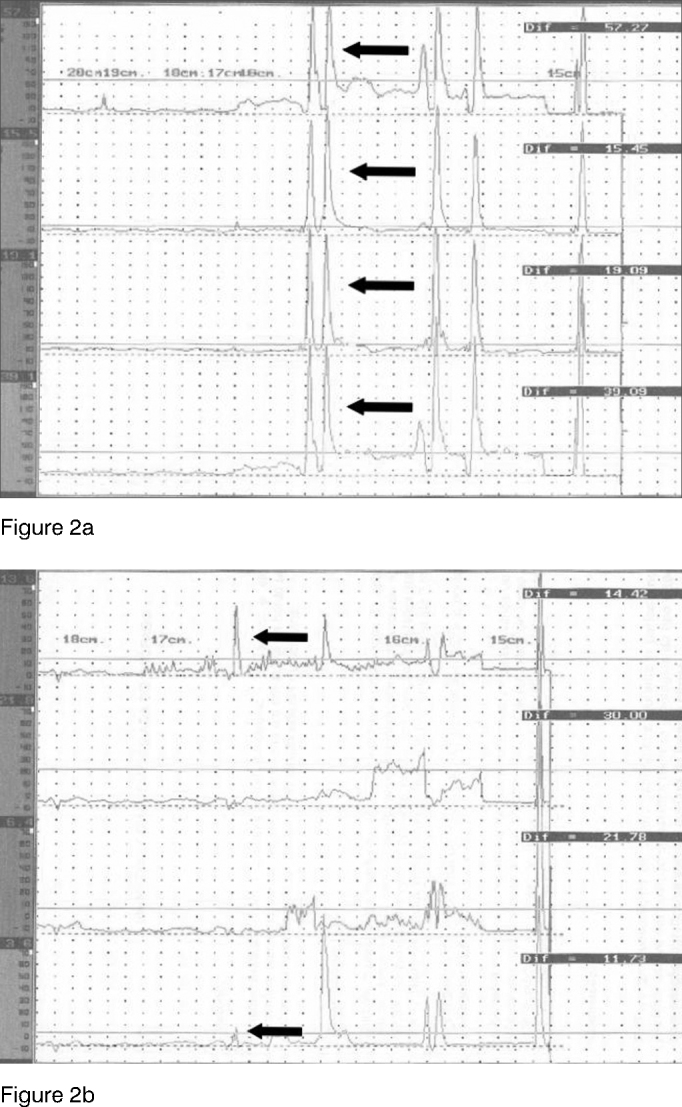
Pressure measurements in the pharyngoesophageal segment for each channel under esophageal manometry before (a) and after (b) botulinum toxin injection. Each base line corresponds to one channel. Pharyngoesophageal segment pressure indication with arrows in each base line.

Manometry findings indicated a reduction in the PES mean pressure after botulinum toxin injection in all eight patients (Table 1 and Fig. 2). Mean PES pressure before BTY injection was 25.36 mmHg. After BT injection the PES mean pressure dropped to 14.31 mmHg (p=0.004).

**Table 1:** Pharyngoesophageal segment pressure under esophageal manometry before (PRE) and after (POST) injecting 100 U of botulinum toxin.

PATIENT	PRE	POST
1	33,0 mmHg	12,2 mmHg
2	17,27 mmHg	12,50 mmHg
3	16,79 mmHg	13,71 mmHg
4	32,7 mm Hg	19,6 mmHg
5	30,0 mmHg	14,1 mmHg
6	16,5 mmHg	13,6 mmHg
7	23,1 mmHg	15,4 mmHg
8	33,5 mmHg	13,4 mmHg

**Figure 3:**
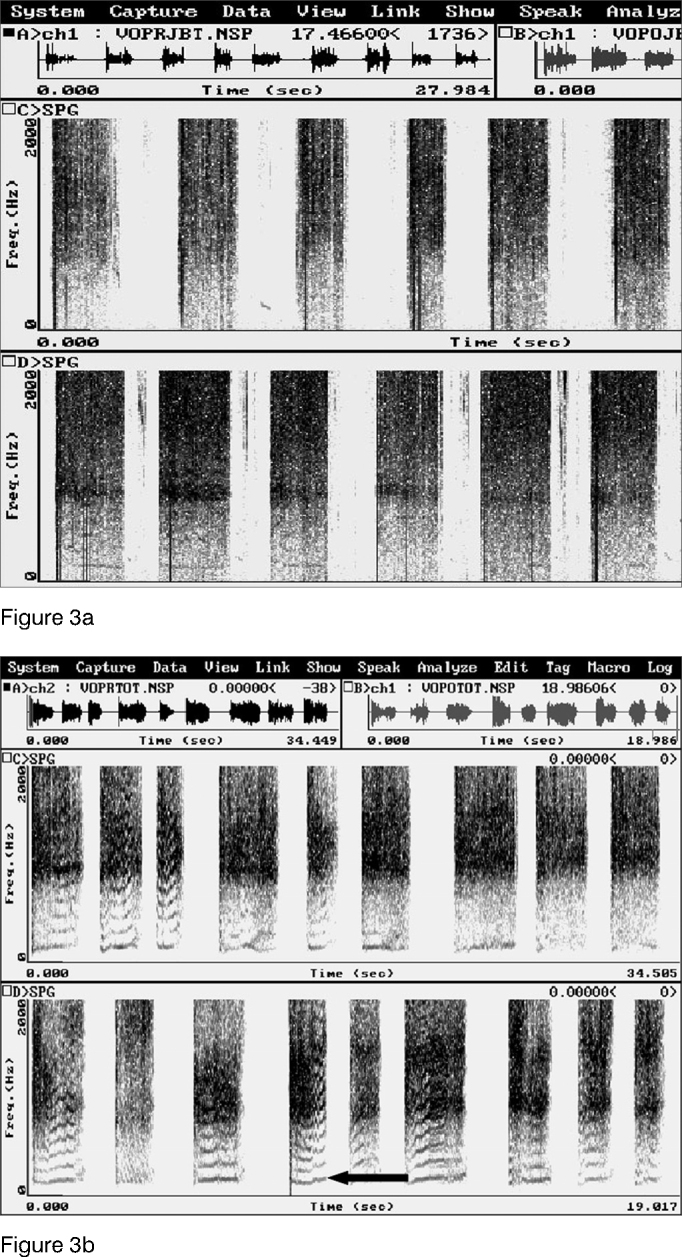
Computerized acoustic tests before (a) and after (b) botulinum toxin injection in the pharyngoesophageal segment. Harmonics observed after botulinum toxin injection (b) not seen before BT injection. Arrow indicating harmonic.

**Figure 4:**
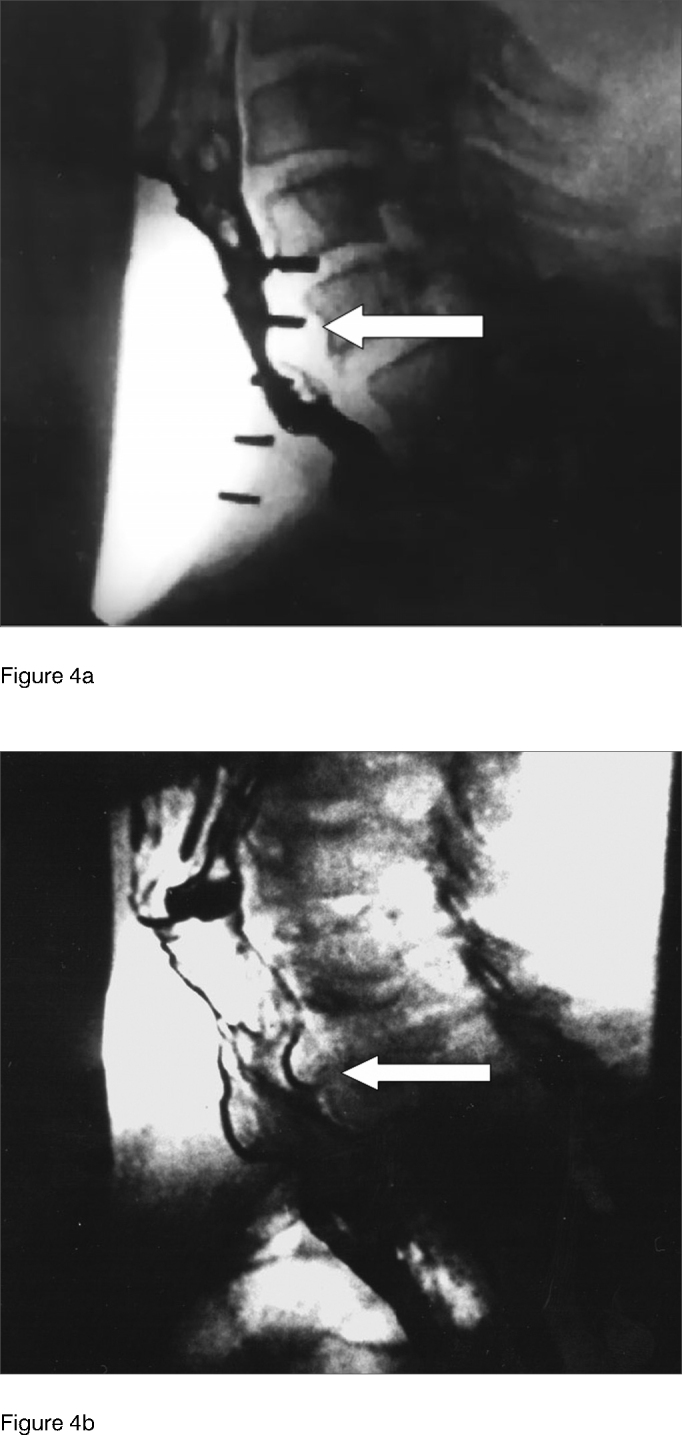
Videofluoroscopy examination showing lateral view during speech before (a) and after (b) botulinum toxin injection on the pharyngoesophageal segment. Arrow indicates spastic area in the pharyngoesophageal segment before injection (a) and spasm-free after injection (b) with increased anteroposterior distance.

Harmonics were identified in a statistically significant manner (p=0.004) in all patients during acoustic analysis after BT injection in the PES (Fig. 3). Before treatment none of the patients could produce harmonics. Effortless voice production was possible in these patients with increased speech time (p=0.004). Before treatment with BT, their speech time was insignificant (Table 2). PES videofluoroscopy during speech showed significant improvements (p=0.004) in PES spasm (Fig. 4) for all patients. No adverse effects were associated to BT use. Clinical improvements in dysphagia were observed in all patients. Patients were followed for 15 to 48 months after BT injection, and none of the patients required additional BT injections.

**Table 2:** Median speech time in seconds before and after injecting 100 U of botulinum toxin in the pharyngoesophageal segment.

Patient	Speech time (s)	
	BEFORE	AFTER
1	1,0	9,0
2	1,0	7,0
3	1,0	7,0
4	1,0	7,5
5	1,0	8,0
6	1,0	8,5
7	1,0	6,8
8	1,0	7,0

## DISCUSSION

PES spasm is a reflex movement triggered by the entrance of air in the esophagus that prevents air from moving into the pharynx. Thus, the pharyngeal mucosa does not vibrate and speech is not possible^1^^,^^3^, ^4^, ^5^^,^^7^, ^8^, ^9^^,^^13^^,^^18^. Spasms can be observed during speech tests under videofluoroscopy^5^^,^^7^^,^^8^^,^^15^^,^^25^ with relaxation during swallowing. In constriction there is no relaxation during swallowing. The treatment in this case is dilation^4^^,^^5^^,^^25^. Spasm is a mechanism devised to protect us against gastropharyngeal reflux, but in TL patients it becomes an obstacle to speech rehabilitation^7^^,^^8^^,^^13^^,^^25^.

BT injections in the PES were initially described in 1994 by Schneider et al.^26^ in the treatment of swallowing disorders with upper esophageal sphincter hypertrophy or hypertonia. The authors used dosages ranging from 80 and 120 units. BT was used initially to treat PES spasm after TEP and introduction of SP by Blitzer et al.^16^. Some authors have shown effects for as long as two years and three months after initial use of BT, with no need of additional injections^18^. A possible explanation is that after the first injection the patients will readapt to the new circumstances^18^.

In primary TEP, myotomy of the middle and lower pharyngeal constrictor muscles is one of the stages of the described surgical approach^12^^,^^27^. Such procedure may be related to increased incidence of postoperative salivary fistulas^8^^,^^12^. Salivary fistulas mean longer hospital stays, higher care costs, delays in speech rehab, later introduction of oral feeding, and delays in postoperative radiotherapy. The actual need for myotomy in TEP is controversial, and ranges from 9% to 79% among TL patients as reported in the literature^1^, ^2^, ^3^, ^4^, ^5^, ^6^, ^7^, ^8^, ^9^, ^10^, ^11^, ^12^, ^13^, as most patients with PES spasm improve spontaneously form this motor disorder six months into follow-up on average^1^. On secondary TEP, myotomy is related to an incidence of salivary fistulas of 10–20%^27^ and the same consequences previously described may occur. The use of BT instead of myotomy to approach patients with PES spasm enables the selection of only the cases requiring PES treatment. Only patients presenting PES spasm are treated, as after six months many improve spontaneously from the condition or even do not see it develop1. BT injections are applied in an outpatient setting with the patient seated and awaken, while the pharyngeal constrictor muscles are monitored through electromyography. This procedure is less expensive that performing a myotomy of the pharyngeal constrictor muscles^17^, and does not present the complications inherent to the myotomy, such as salivary fistula and PES hypotonia, the latter lacking a definitive solution and leading to hypotonic speech. One should bear in mind that even after performing a myotomy on the medial and lower pharyngeal constrictor muscles spasms may occur as the muscle fibers draw closer to each other again^1^^,^^7^^,^^10^^,^^11^^,^^17^; botulinum toxin injections may then also be used.

Speech time evaluation is an indirect method to assess PES spasm in patients rehabilitated with TES and SP7. When speech time is under eight seconds, the patient may have PES spasm^7^. In this study we observed that all patients with PES spasm improved after BT injection as seen in videofluoroscopy examination. PES pressure was reduced as observed in esophageal manometry tests, and improvements were observed in speech time.

As all patients with TES and SP had marked alterations in their speech when compared to laryngeal speech, the only parameter left for evaluation was presence or absence of harmonics under computerized acoustic analysis. As patients with spasm could not speak, they could not produce harmonics. After PES BT injection and improving from spasm, all patients could speak, consequently producing harmonics as observed in computerized acoustic tests.

Objective evaluation of PES spasm can be done through measurements of intratracheal pressure, in which levels greater than 40cm of H_2_O may trigger spasms^18^. The modified insufflation test can also be used with the same purpose, in which pressures above 20 mmHg^10^^,^^15^ indicate spasm. Using videofluoroscopy with image digital analysis also allows the measurement of spasm during speech. Esophageal manometry is another objective method to analyze PES relaxation after BT injection in the rehabilitation of TL patients with spasm. Total laryngectomy patients have median PES pressures lower than that of individuals with larynx^28^. A previous study observed that median PES pressures after neurectomy of the pharyngeal plexus for the treatment of spasm resulted in statistically significant pressure reduction and found that pressure values greater than 20 mmHg can be used to separate patients with spasm from patients without spasm^29^. In this study we looked objectively into PES intraluminal pressure before and after BT injection. Reductions in PES pressure under esophageal manometry were correlated to improved speech quality. Pressure levels were reduced in all patients. Computerized esophageal manometry is an objective method that can be used in the assessment of patient response to PES spasm treatment after BT injection in TL patients, rehabilitated with TES and SP.

## CONCLUSION

Computerized esophageal manometry is an objective method that allows the quantification of PES intraluminal pressure and may be viable in the analysis of the effect of BT injections in this site.

Statistically significant (p<0.05) PES pressure reductions observed under esophageal manometry were seen in all patients after PES BT injection.

All patients had increases in their speech time values after BT injection (p<0.05).

All PES spasm patients who took BT injections presented PES pressure reduction and improved spasm under videofluoroscopy (p<0.05).
